# Facial width-to-height ratio differs by social rank across organizations, countries, and value systems

**DOI:** 10.1371/journal.pone.0187957

**Published:** 2017-11-09

**Authors:** Tim Hahn, Nils R. Winter, Christine Anderl, Karolien Notebaert, Alina Marie Wuttke, Celina Chantal Clément, Sabine Windmann

**Affiliations:** 1 Department of Psychology, Cognitive Psychology II, Johann-Wolfgang-Goethe University, Frankfurt, Germany; 2 Research Center of Marketing and Consumer Science, Katholieke Universiteit, Leuven, Belgium; Macquarie University, AUSTRALIA

## Abstract

Facial Width-to-Height Ratio (fWHR) has been linked with dominant and aggressive behavior in human males. We show here that on portrait photographs published online, chief executive officers (CEOs) of companies listed in the Dow Jones stock market index and the Deutscher Aktienindex have a higher-than-normal fWHR, which also correlates positively with their company’s donations to charitable causes and environmental awareness. Furthermore, we show that leaders of the world’s most influential non-governmental organizations and even the leaders of the Roman Catholic Church, the popes, have higher fWHR compared to controls on public portraits, suggesting that the relationship between displayed fWHR and leadership is not limited to profit-seeking organizations. The data speak against the simplistic view that wider-faced men achieve higher social status through antisocial tendencies and overt aggression, or the mere signaling of such dispositions. Instead they suggest that high fWHR is linked with high social rank in a more subtle fashion in both competitive as well as prosocially oriented settings.

## Introduction

An increasingly researched measure of facial appearance is the width of a face in relation to its height (facial Width-to-Height Ratio, fWHR). Empirical studies have consistently linked the measure with antisocial behavioral tendencies in a large number of well-controlled studies. For example, men with high fWHR were described to be more aggressive [1–4], more fearless-dominant [5], higher in psychopathy [6], and less likely to die from direct physical violence than narrower-faced males [7]. Wider faced men are more willing to cheat in order to increase their financial gains, more readily exploit the trust of others, and more often explicitly deceive their counterparts in a negotiation [5, 8–10]. Observers perceive and interpret high fWHR as a signal of untrustworthiness, and down-regulate their level of cooperation accordingly [8], potentially leading to self-fulfilling prophecies [11].

In view of the suggested social traits, individuals with relatively larger fWHR should be at a higher risk for social rejection, discrimination, and even ostracism, as holds generally for antisocial and uncooperative individuals [12]. Surprisingly, however, research shows fWHR to be linked positively with success and goal attainment in various competitive social contexts. For instance, fWHR has been demonstrated to positively correlate with the financial success of corporate leaders [13]; for evidence that this association might only hold for simple-structured companies, see [14], with the achievement drive of past US presidents [15], and with team sports performance [16, 17]. Furthermore, recent studies have shown wider-faced men to be preferred short-term mating partners of female volunteers [18], and to more successfully reproduce in general [19].

One way to explain this apparent paradox is that the social contexts in which the indicators of success were observed so far were distinctively competitive in nature (economy, politics, sports, mating) so that egoistic, dominant, and aggressive behaviors may overshadow any agreeable or prosocial tendencies. Another explanation would be that the existing results are of limited generalizability: They may hold only for physical activities, cf., [16, 17] simple-structured groups [14], or other specific subgroups of individuals (e.g., low ranking males) while bearing little relevance for public life in large-scale human societies.

To elucidate the overall relevance of fWHR for social status in public life, we performed a series of studies in highly prominent males using solely publicly available data. We investigated, first, the link between fWHR and social position in these individuals, thereby addressing the ecological relevance of a potential link between fWHR and social rank; and second, the role of competitive as opposed to explicitly prosocial value systems in mediating this link to specify the generalizability of the relationship with regards to social context. The full dataset is available for download (see S1 File Supplementary Information).

To pursue the first question of public-life relevance, we determined whether a positive association exists between fWHR as taken from publicly available portrait photographs and social rank in some of the world’s highest ranking individuals. Specifically, we compared fWHR of male, Caucasian CEOs of all companies listed in the Dow Jones stock market index and of those listed in the Deutscher Aktienindex (DAX) with control data collected from a large number of publicly available databases (total *N* = 392), thereby providing a strong norm reference data set. However, since CEOs were on average older than were the control individuals and smiled more on the photographs, we used one additional database that contained portrait photographs of men across all ages with both neutral and happy facial expressions to determine the strength of the influence of age and smiling expression on the fWHR measure, and to carefully match the comparison groups on these variables.

To address our second question of whether any link between fWHR and social rank may be bound to competitive and individualistic (as opposed to cooperative) environments and value systems, we chose two approaches: First, we related fWHR of the Dow Jones CEOs to prosocial performance measures, namely, first, to their popularity and likeability as rated by their employees, and secondly, to records of their companies’ charitable donations and environmental conscientiousness, again using only publicly available data.

However, we reasoned that although the indices appear to quantify prosocial intent, they still refer to the leaders of profit-oriented organizations operating in a context of intense between-group competition. In this particular environment, dominant leaders may be liked better, may be more successful, and may display more prosocial gestures such as charitable donations and environmental sustainability practices not because they favor prosocial values, but as a strategic means of promoting the companies’ reputation and image, with the ultimate goal to increase turnover, and with it the leaders’ personal benefits and salaries. Therefore, we pursued an additional approach: To determine whether the relationship between fWHR and social rank also holds for leaders of organizations who are not profit-seeking in the first place, but declare to serve prosocial, humanitarian values, without any overt self- interest in economic payoff, we also investigated leaders of non-governmental organizations (NGOs) and of the Roman Catholic Church (popes).

## Materials and methods

### Data sampling

#### Facial width to height ratio

FWHR was measured as described in [16] by two raters (Rater 1 and Rater 3) using a software ruler. Specifically, the open-source software ImageJ (imagej.net) was used to measure the distance between the lip and brow (height of upper face) and the left and right zygion (bizygomatic width) of the images. From these two values, we then computed facial width to height ratio.

Twenty-four pictures of CEOs of DOW Jones and 31 of DAX indexed companies’ CEOs were obtained from publicly available websites and pictures were included if two raters—, one of whom was blind to the study goals—agreed on their quality. Using other stock market indexes including a larger number of CEOs did not appear advantageous as a very large number of these CEOs would be non-Caucasian. In addition, for companies not as prominent as those listed for example in the Dow Jones Index, it is extremely difficult to obtain recent portrait photographs of sufficient quality. Notably, all selected photos were strictly taken from a frontal perspective. In many instances, photos of NGOs and popes appeared not specially made or selected for publication on personal websites or for promotion purposes but appeared incidental.

CEOs of non-governmental Organizations were selected according to *The Global Journal*: *The Top 100 NGOs*, http://theglobaljournal.net/top100NGOs/). Only pictures of male Caucasian NGO leaders were used which resulted in 48 pictures.

All popes of the Roman Catholic Church whose photographed portraits were publicly available on the Internet in sufficient quality were used (9 out of the 12 popes since 1848).

Pictures of normal controls were taken from BaDaDa Face Database (http://uni-bamberg.de/allgpsych/), BioID Face Database (http://www.bioid.com/index.php?q=downloads/software/bioid-face-database.html), CK+ Face Database [20], CVL Face Database (http://www.lrv.fri.uni-lj.si/facedb.html), FACES Face Database [21], Georgia Tech Face Database (http://www.anefian.com/research/face_reco.htm), MIT Face Database [22], Psychological Image Collection at Stirling (Aberdeen Face Database, Nottingham 2 Face Database, Stirling Face Database, Utrecht Face Database; pics.stir.ac.uk). One rater (Rater 1) measured fWHR for all pictures. One further rater (Rater 2)–blind to the goals of the study and the leadership status of the pictured individuals—measured fWHR of all leaders and the additional control group used for the matching (the database of [23]). Interrater reliabilities for the subgroups ranged between *r* = .86 and *r* = .94 (Spearman). Means of the measurements from Rater 1 and Rater 3 (*r* = .89) formed the basis for all analyses involving the large control sample. Means of the measurements of Rater 1 and Rater 2 (*r* = .90) formed the basis for all analyses involving the matched control samples, except for the NGO group, where only measurements of Rater 1 were available for both comparison groups.

#### Correlational analyses

For all correlations with external (“prosociality”) measures, we ensured that the Dow Jones CEOs had been in office for at least one year before the external measure was taken. Since the measures were taken from the year 2012, this means that CEOs involved in the analyses had been in office at least since 2011 (or earlier).

We obtained data regarding employee satisfaction with their CEO and their company through the website “Glassdoor.com”. Glassdoor is an independent job and career community where employees can rate their CEOs and workplaces. Information on employee satisfaction with their companies from 21 of the 24 Dow Jones companies was entered into our analysis as three CEOs had not been in office for at least one year. We obtained the percentage of employees that approved of their CEO. These percentages are based on a substantial number of reviews: For employee satisfaction with company, a median of 392 employees (between 78 and 3108) had submitted a rating. For employee satisfaction with the CEO, a median of 1232 employees (between 162 and 5672) had submitted a rating (http://www.glassdoor.com/Reviews/company-reviews.htm).

As an environmental engagement and corporate sustainability index, we used the Newsweek Green Ranking, obtained through Newsweek’s homepage (www.newsweek.com). Since Newsweek uses performance data from the year 2011 to create the Green Ranking 2012, we again excluded three CEOs that had not been in office for at least one year. All remaining 21 Dow Jones companies headed by male CEOs were listed and their green ranks for the year 2012 were collected. A low number (high rank) represents a more ecological and more transparent company (for details, see http://www.newsweek.com/2012/10/22/newsweek-green-rankings-2012-global-500-list.html).

Corporate charitable donations of 19 of the Dow Jones companies were obtained for the year 2012 through “The Chronicle of Philanthropy” (http://philanthropy.com/article/How-America-s-Biggest/140269/#id=101005). The donations of three companies were not listed and two CEOs had not been in office for at least one year. We acquired each company’s revenues 2012 from fortune.com which annually releases the Fortune Global 500 list (http://fortune.com/fortune500/).

### Data analysis

#### Inferential statistics

Differences between the control groups and the groups of leaders were tested for significance using two-sample t-Tests. As the distribution of the data was not always normal, we opted for a permutation-based approach conducted as follows: First, we ran the t-Test with permuted data 10,000 times, thereby creating a distribution of t-values under permutation. Significance was then computed by counting the number of times that the true, absolute t-value (without permutation) was smaller than or equal to the absolute values of the t-value distribution under permutation and dividing this number by 10,000. Note that using a standard t-Test approach does not substantially change the results. Two-tailed p-values are reported for all significance tests for illustrative purposes, but 95% bias-corrected and accelerated confidence intervals (CI) are shown and reported for all group differences involving fWHR.

Correlations were assessed in an analogous manner as follows: First, we computed a correlation with permuted data 10,000 times thereby creating a distribution of r-values under permutation. Significance was then computed by counting the number of times that the true, absolute Spearman rank correlation r-value (without permutation) was smaller than or equal to the absolute values of the r-value distribution under permutation and dividing this number by 10,000. Again, applying a classic approach for significance testing practically did not change the results.

#### Confounds

When comparing our large control sample to the CEO groups, we noticed a potential difference in facial expression and age. Different from controls, many CEOs smiled on the photographs, and they also appeared to be older on average. To assess whether controls and CEOs indeed differ regarding facial expression and age, we ran another study. Two additional raters, blind to the study goals, were trained to rate the smile on more than 100 photographed faces taken from an independent database [23]) using a five-point Likert scale (0 to 4). The experimenter (SW) met with one rater, and used unrelated portrait photographs for practice at first. The following procedure was agreed upon: Rating 4 was given for a broad smile with open mouth and/or visible teeth; rating 3 for a broad smile with closed lips that appeared happy and content, rating 2 for a clear smile that appeared to communicate friendly attitude, rating 1 for a polite or barely visible smile, rating 0 for no smile or neutral expression. About 100 photos were discussed before the rater went on to convey the procedures to the second rater. The two then independently rated the smile of the Dow Jones CEOs, the Dax CEOs, the NGO CEOs, the popes and the entire control sample. The Minear and Park faces [23] were used as controls for this analysis for their wide age range and the inclusion of happy facial expressions. Mean fWHR of this sample (*M* = 1.856, *SD* = .135, *N* = 114) was very close to that of the large sample of control faces (*M* = 1.865, *SD* = .134, *N* = 392, *t*(504) = .560; *p* = .576, Rater 1 only). As interrater reliability for the smile rating was high (Spearman *r* = .83, *p* < .001), the mean smile rating of both raters was used in the subsequent matching procedures.

Results confirmed our observations that CEOs smiled more on average than did controls. Differences to controls were significant for the Dow Jones CEOs (*t*(136) = 2.64, *p* = .009), the Dax CEOs *t*(143) = 2.93, *p* = .005), and the NGO CEOs (*t*(150) = 3.63, *p* < .001). The popes did not differ from the control sample in this manner (*t*(121) = .016, *p* = .9417). However, mean age of the control sample also differed, at least *trend*-wise, It was lower than that of the Dow Jones CEOs (*t*(136) = 2.13, *p* < .035) and the Dax CEOs (*t*(143) = 1.70, *p* < .092). For the popes, age was not available; and age of the NGO CEOs was available only for 9 of the 38 cases.

We observed in the control group that measured fWHR was indeed significantly correlated with both, rated smile (Spearman *r* = .299, *N* = 102, *p* < .001) and age (Spearman *r* = -.302, *N* = 102, *p* < .001). Thus, smiling increases and age decreases the fWHR measure; with only the latter having been reported before [24]. Thus, to ensure that our group comparisons were not distorted by these two confounds, we performed two types of control analyses.

#### Conventional matching procedure

We selected control faces from the database (Minear and Park, 2004) in such a way that the resulting means in rated smile and age were very close to those of the DAX, DOW Jones and NGO CEO groups, while maintaining as many faces as possible. To this end, we selected all faces for which at least one of the two raters had given a rating above 0. For six faces that appeared twice in the database, we maintained only the one that was smiling more. Then, to increase age in the control group, we added data of individuals above the age of 80 (regardless of expression) and deleted individuals below the age of 20 (regardless of expression). This resulted in a “CEO-matched control group” of *N* = 57, mean age 55.00 years (CI [48.44 61.45]), mean smile 1.43 (CI [1.12 1.75]); compared to DAX (*N* = 31, mean age 56.03 years, CI [54.14 58.08]; *p* = .77); mean smile 1.40, (CI [1.09 1.71], *p* = .91), DOW Jones (*N* = 24, mean age 59.25 years, CI [57.83 60.63], *p* = .41), mean smile 1.41, (CI [1.04 1.81], *p* = .96), and NGO CEOs (*N* = 38, mean age unknown, mean smile 1.50, SD = .95, *p* = .75).

The popes were substantially older (with an estimated mean age above 70 years) than were the large control and all CEO groups, with most of them showing a neutral facial expression, so that, strictly speaking, the large control group appeared appropriate for comparison, with the markedly higher age of the popes decreasing the chance of finding a significant difference in fWHR. We nonetheless created a “pope-matched control group” from a suitable database [23] using faces of individuals older than 40 years with a smile rating of 3 or less. This resulted in *N* = 42 controls with a mean age of 70.45 years (CI [66.42 74.25]) and a mean smile of 0.94 (CI [0.62 1.29]) compared to the popes (*N* = 9, age estimated around 70 years, mean smile 0.78, CI [0.33 1.21], *p* = .67).

#### State-of-the-art matching

Generally, the goal of any matching procedure is to achieve an equal, multivariate distribution of covariates within the matched experimental and control groups. When exact matching of covariate distributions cannot be achieved—as is the case for virtually all empirical investigations—best practice holds to 1) minimize imbalance for all covariates and 2) adjust for any remaining differences [25], thereby creating a “doubly robust” approach. Although often used, matching covariate means, as done above, is thus insufficient. Importantly, using significance tests to assess equality of means is not advisable in the first place and the magnitude of mean differences does not allow for an estimation of the bias related to the covariates (for a more detailed introduction to this topic, see [26]. In order to obtain an optimally similar, multivariate distribution of covariates, we used *MatchIt* software [27] to select a matching subset of control faces employing the *Optimal Matching Procedure* which minimizes the average absolute distance across all matched pairs. Then, potentially remaining differences in covariate distributions were modelled using standardized distances between the remaining matched samples in covariate space. Specifically, we assessed the significance of the difference in fWHR between the matched control group and the respective CEO group in an Analysis of Covariance (ANCOVA) framework using rated smile, age, and standardized distances between the samples as covariates. Indicating successful matching, neither age nor rated smile was significant after the procedure (Dow Jones CEOs: all p>.52; DAX CEOs: all p>.33).

#### Prototypical faces visualization

For visualization purposes, we created an average morph for CEOs of DAX and Dow Jones companies as well as for the popes, the heads of NGOs and all control groups using Abrosoft Fantamorph 5 Software (www.fantamorph.com).

## Results

**Fig 1:**
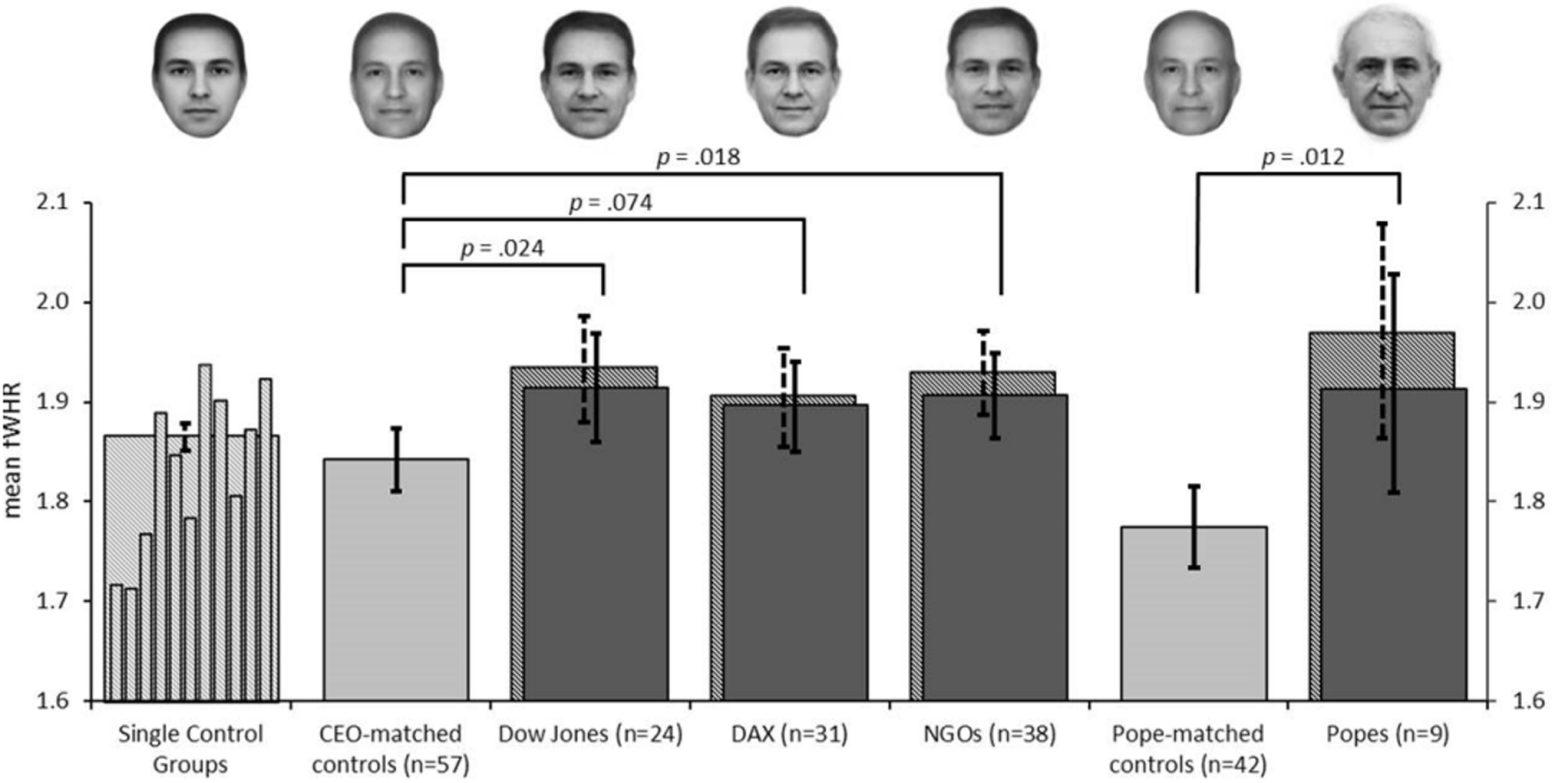
Mean facial width-to-height ratio (fWHR) of all analyzed groups. Plainly shaded bars indicate measurements by Raters 1 and 2; patterned bars indicate measurements by Raters 1 and 3. The main analysis compares CEOs, NGOs and popes (patterned, dark grey) with the mean of all control groups (patterned, light grey). The matching analysis compares CEOs, NGOs, and popes (plain, dark grey) with their matched controls (plain, light grey), respectively. Individual control groups (patterned, light grey, thin bars) from left to right: MIT (*N* = 6), BioID (*N* = 13), Stirling (*N* = 15), BaDaDa (*N* = 22), GTdb-crop (*N* = 26), Utrecht (*N* = 28), CK+ (*N* = 31), FACES Database (*N* = 40), Aberdeen (*N* = 53), Nottingham2 (*N* = 63), FaceDB (*N* = 95). Error bars indicate 95% confidence intervals.

We found fWHR in the photographs of both the DOW Jones (mean difference .069, CI [.014 .125], *p* = .014, Hedges g = .51) and the DAX CEOs (mean difference .041, CI [-.007 .092], *p* = .094, g = .30) to be higher than that of the large control group (see Fig 1), although the difference remained below significance for the DAX group. Importantly, using the conventional matching procedure, the results for the Dow Jones group remained essentially unchanged when controls were matched to the CEOs for age and facial expression (mean difference .072, CI [.009 .135], *p* = .024); results for the DAX CEOs remained marginally significant (mean difference .055, CI [-.002 .110], *p* = .074). A morphed picture of the CEO groups and their age- and expression-matched controls is included in Fig 1. Also, the complementary multivariate matching procedure produced comparable results: The differences in fWHR all remained significant after sample matching and bias correcting in the ANCOVA framework (Dow Jones CEOs vs. controls: *F*(1,43) = 5.24, *p* = .027, partial η^2^ = .11; DAX CEOs vs. controls: *F*(1,57) = 4.72, *p* = .034; partial η^2^ = .08).

**Fig 2:**
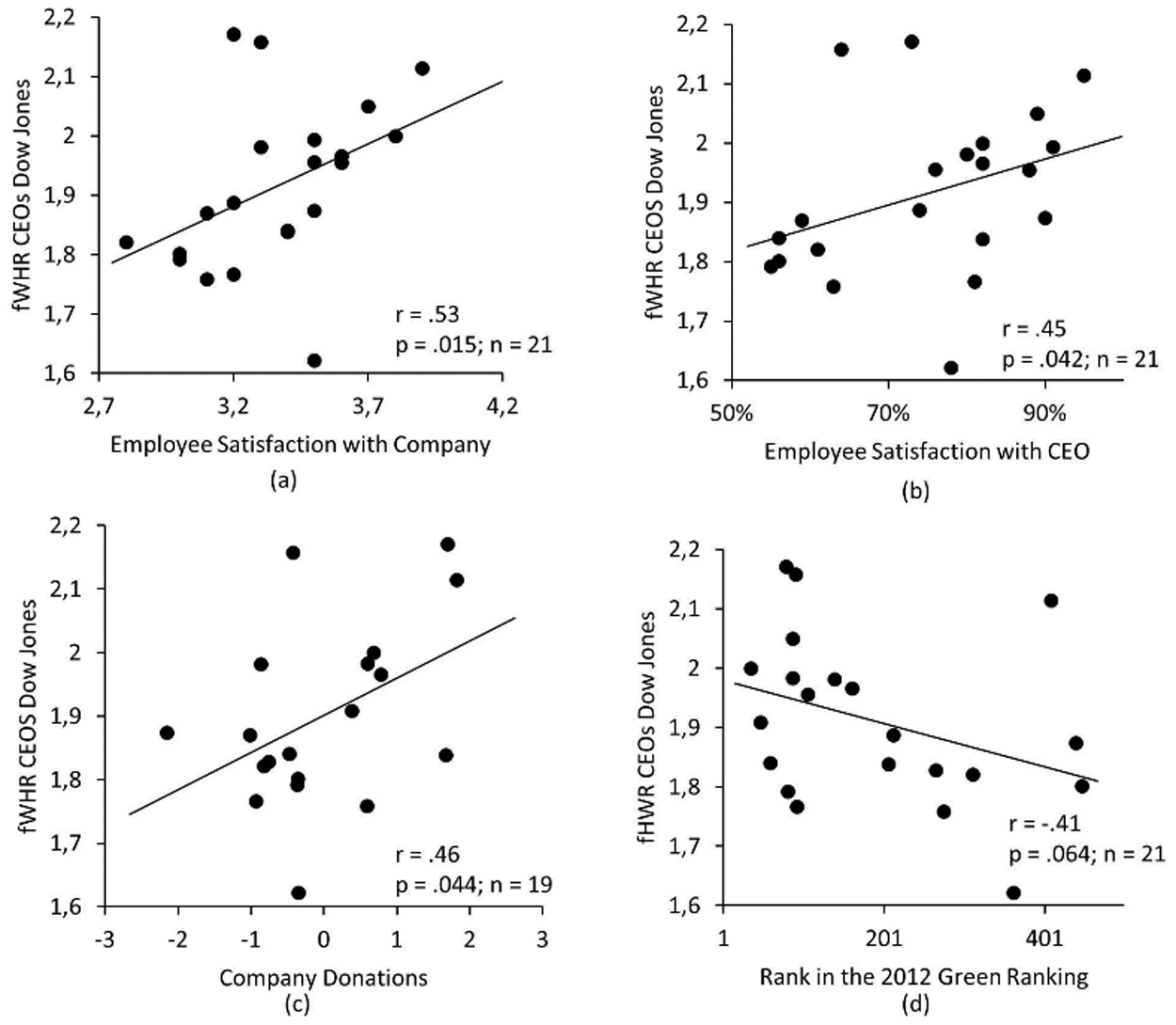
Scatter plots showing the associations between facial width-to-height ratio of CEOs of companies listed in the Dow Jones stock market index and performance measures. a) Employee satisfaction with their companies, b) employee satisfaction with their respective CEOs, c) the companies’ charitable donations and d) the companies’ positions in the 2012 Green Ranking (for additional analyses controlling for company revenues, see main text).

To address our second question of whether the link between fWHR on the published photographs and social rank may be bound to competitive and individualistic (as opposed to prosocial and cooperative) social value systems, we assessed the association of fWHR in the photographs of the leaders on the one hand and measures of their prosocial engagement and popularity on the other. We found that fWHR of the Dow Jones CEOs’ portraits correlated significantly with both, the overall satisfaction score of their employees (*r* = .53, *N* = 21, *p* = .015, Fig 2a), and the percentage of employees approving of their CEO (*r* = .45, *N* = 21, *p* = .042, Fig 2b). Note that these associations were not significant when controlling for companies revenues (overall satisfaction: *r* = .38, *N* = 16, *p* = .167; percentage approval: *r* = .31, *N* = 16, *p* = .164). In addition, fWHR of the Dow Jones CEOs’ photographs was positively associated with their companies’ charitable donations (*r* = .49, *N* = 19, *p* = 0.033; this result remained significant when controlling for the companies’ revenues: *r* = .46, *p* = .044, Fig 2c). Finally, a similar (although only marginally significant) association was observed for the ranking of their companies in environmental sustainability as expressed in the *Newsweek Green Ranking* (*r* = -.41, *N* = 21, *p* = .064; Fig 2d). Note that most of these correlations became stronger when only CEOs were considered who had been in office before 2010 (so that they had been in office at least three years), namely *r* = -.45 (*N* = 17) for Green Ranking, *r* = .42 (*N* = 14) for corporate givings, *r* = .79 (*N* = 14) for overall employee satisfaction, and *r* = .75 (*N* = 14) for percent satisfied with CEO. However, due to the small sample size, we have not performed any significance tests for these correlations.

While these correlations are only indirect measures of the CEOs’ social motives, they challenge the stereotypical interpretation that wider faces in CEOs speak for higher self-interest overall, with little regard for social impact, relative to their smaller faced coevals. On the other hand, we reasoned that some of the indices we used in the correlational analyses may serve selfish goals indirectly, particularly by attracting customers and partners and by enhancing the companies’ reputation, which will eventually lead to greater profits. Hence an ultimately selfish interest may lie at the bottom of any of these performance measures. To determine whether the relationship between fWHR and social rank also holds for non-profit seeking organizations whose explicit purpose, from the outset, is to serve prosocial, humanitarian values, we investigated published portraits of leaders of two further groups, first, CEOs of non-governmental organizations (NGOs), and second, the popes of the Roman Catholic Church.

Compared to the large control sample, we found higher fWHR in published portraits of both, the NGO CEOs (mean difference .065, CI [.012 .118], *p* = .006, *g* = .48), and the popes (mean difference .104, CI [.002 .219], *p* = .023, *g* = .77; Fig 1). Compared to the “CEO- matched control group” generated on the basis of conventional procedures, NGO CEOs still showed significantly higher fWHR (mean difference .068, CI [.013 .122], *p* = .018). Likewise, fWHR of the pictured popes was higher than that of the “pope-matched controls” (mean difference .139, CI [.027 .246], *p* = .012). Again, the multivariate matching approach led to highly similar results: Matching for rated smile only (as age at the time when the picture was taken was available for none of the popes and only for 9 of the 38 NGO CEOs) in the NGO CEO group and the popes led to comparable results (NGO CEOs vs. controls: *F*(1,72) = 4.31, *p* = .041, partial η^2^ = .06; popes vs. controls: *F*(1,14) = 3.70, *p* = .036, partial η^2^ = .21).

Overall, across all leaders, fWHR is larger in the CEO-group as a whole (Dow Jones, DAX, popes, and NGOs combined, *N* = 102) compared to the large control group (*N* = 435; *t*(492) = 4.10, *p* < .001, *g* = .46).

## Discussion

We showed higher fWHR in published portrait photographs of successful leaders of different types of organizations across different countries and social value systems. Our analysis included portraits of the CEOs of top companies in the USA and Germany as well as heads of some of the largest and most popular non-profit organizations, and even the popes as leaders of one of the largest religious groups in the world. For the Dow Jones Index CEOs, we furthermore showed that these men’s pictured fWHR correlates significantly with their companies’ donations to charitable causes, and marginally significantly with their environmental engagement. It furthermore correlates significantly with overall satisfaction and likability ratings of their employees. This evidence powerfully illustrates the predictive value (statistically, not causally) of fWHR obtained from published portraits for successful leadership, but at the same time warrants against the potentially discriminating notion that wider faces uniformly represent antisocial, aggressive, and deceptive traits. Quite to the contrary, our results suggest that at least in the public eye, in view of their large-scale societal impact, wider faced leaders impress as likable and socially responsible individuals.

Taken at face value, our results suggest that leaders of both profit- and non-profit seeking organizations may indeed have wider faces than individuals at lower social ranks, extending earlier results on company’s CEOs [10, 14, 13, 28]. Under this interpretation, linking fWHR to testosterone exposure during puberty may help to explain the underlying mechanisms [29, 30], but see [31], while taking into account that common beliefs about the effects of testosterone are full of stereotypes themselves [32]. On the one hand, the hormone has indeed been associated with (reactive forms of) aggression (reviewed in [33, 34]), but on the other hand, recent studies have shown that at least in some contexts, testosterone can actually increase fairness [32] and cooperation [35–37], as well as reduce lying [38], particularly when there is no threat of competition [37, 39]. In a similar vein, research on individuals with psychopathic traits has begun to describe socially adaptive lifestyles [39] and subtle forms of strategic defection that can facilitate ascent in social rank [40]. Like testosterone, fWHR has been associated with psychopathic personality traits [5, 6], but has also been found to correlate positively with “self-sacrificing” under conditions of group competition [41]. Adding our results, when interpreted in terms of fWHR-associated personality traits, the emerging pattern suggests that any black-and-white type of thinking about the behavioral implications of either high fWHR or the effects of testosterone seems inappropriate. Especially when considering the NGO CEOs and the popes − individuals who publicly portray themselves as devoted to humanity and to solving global problems—again only a face value interpretation—we suggest that men with high fWHR are well able to commit to prosocial goals and cooperation. However, quite obviously, they can at the same time preserve their aspiration to dominate and to strive for social status. This mixture of seemingly contradictory motives may not be easy to understand from the standpoint of classic philanthropy, but it likely exists in human behavior and would need to be reconsidered in sociobiological theories, whether or not these make reference to facial morphology.

Importantly, although the testosterone-related interpretation of our findings is in line with the literature, there are two plausible alternatives. One is that fWHR—rather than implicating leadership-prone behavioral dispositions—may trigger perception of such dispositions in observers. Because our results bear no causal implications for the proposed relationship between fWHR and social rank, they do not discriminate between a behavioral account (potentially testosterone-mediated) and an attributional account. From the perspective of the latter, men with higher fWHR might be more likely to be selected as leaders because fWHR is taken (consciously or unconsciously) as a signal for behavioral dispositions thought to underlie successful leadership.

In fact, some recent studies particularly on facial appearance of CEOs speak in favor of this account. One study [13], quite comparable to ours in design, showed that the higher-than normal fWHRs of CEOs of UK Financial Times Stock Exchange Index (FTSE) companies correlate with observer’s ratings of dominance and successfulness, suggesting that people expect CEOs with high fWHR to perform well. Another quite sophisticated study investigated fWHR in successors of CEOs who have been dismissed after their companies became known to be involved in financial misconduct [42]. The successor CEOs were found to have *lower* fWHR compared to CEOs who were newly appointed for other reasons, presumably because narrower-faced CEOs signal more trustworthiness and integrity. The positive impact of the company’s “facelift” was also reflected in reactions of analysts and the media to the restatement, as shown on the basis of computer-aided text analysis. Other authors speak in favor of the selection account because they find a conglomerate of facial features (including fWHR) to statistically predict CEO status, but not CEO performance [28]. Because fWHR is never the only feature a face displays, such multivariate approaches can help greatly to advance the field, although they cannot directly solve the question of causality.

Taken together, there is ample evidence suggesting that wider-faced men are indeed perceived differently. The perceptions they trigger, and the reactions they receive, may in turn shape their counter-reactions and expectations [4, 43]. These are the kinds of recursive processes that typically arise from stereotyping.

What would be needed to identify the starting point of the spiral are longitudinal or specially designed experimental studies. In the absence of such evidence, which is difficult to obtain particularly from the world’s top leaders involved in the present study, we can only speculate whether signaling mechanism alone can be strong enough to account for our results, especially with regards to the correlations we obtained between fWHR and external performance measures. These correlations have been determined within the group of the Dow-Jones Index CEOs, where the variance in fWHR is much smaller than between CEOs and controls. It’s a long way for observers from detecting such subtle differences in the fWHR of CEOs to linking these differences to the corresponding differences in behavior, indexed by charitable donations and environmental initiatives.

In addition, contrary to the idea of a “Great Man”, leadership in a complex, modern world can hardly be understood as a unidimensional concept [44]. What signals a good leader depends in large parts on context, task goals, and follower attributes [45, 46]. The same is true for prosociality; it is not a unitary construct. For example, cooperation within a group may serve competition with other groups, and it is precisely this context of between-group competition in which men with high fWHR have been shown to be particularly cooperative [35]. Thus, it may be more appropriate to think of fWHR-related traits in terms of social dominance and competitiveness, or perhaps “strategic” and “flexible” forms of prosociality (pursuing social status rather than ultimately prosocial goals) than in terms antisocial traits. Such understanding may help to resolve the paradox that men with high fWHR are perceived and described as successful [13, 18] despite the evidence of their higher aggressive potential [1–4]. Nonetheless, for any such multi-faceted and context-dependent behavioral traits, it will be much harder for observers to find a reliable physical marker than it were for a consistently salient and unidimensional attribute such as “aggression” [41].

Our final argument against a pure signaling/attributional account is that it leaves open the question of how observers come to take fWHR as a cue for leadership proneness in the first place. If these heuristics were entirely invalid, leading to false predictions more often than not, then it is hard to imagine why observers would develop and maintain them. Thus, taken together, we believe that fWHR is indeed associated with leadership-related motivational traits such as social dominance and strive for social impact, at least to some degree, above and beyond the signaling of such dispositions, but we note that our data pattern does not allow for any directional claims.

A third possibility for interpretation of our data, besides the behavioral trait account and the signaling account, stems from the fact that the pictures of the leaders in this study were published on the internet, often purposefully selected with the intent of proper representation, whereas pictures of controls were taken and published for research purposes. Most likely, many of the company’s CEO photographs were taken by professional photographers, perhaps using special camera technology, angle or lighting, and some of them may have undergone additional picture editing. Quite plausibly, they were selected and designed to match the preferred personal image of the leader in many cases (although from our observations, we find this less likely for the NGOs and the popes than for the company’s CEOs). Along the way, facial width may have been artificially pronounced and augmented (with or without intent) [47]. If such artificial, technical factors are the cause for the wider faces, then the interesting question becomes why leaders would arrange for, select, or otherwise favor wider-than-normal facial portrayal in support of their preferred public image instead of average or even smaller faces. Again, the most plausible explanation is that wide faces are intuitively thought to imply and/or signal motivational traits that predispose for successful leadership, with all its multifaceted implications for social behavior.

Overall, given our correlational design, we cannot draw any causal inferences from our results. We therefore cannot estimate in how far behavioral traits, impression-management strategies, observer’s attributions, or any combination of these, may have mediated the reported relationships [13, 28, 42]. With public data gathered online, it is tricky to address questions of causality. For that same reason, any assumption of higher prosociality in the trait motivation of NGOs and the popes compared to the DAX and DOW Jones CEOs is speculative. We have no direct information about these leaders’ underlying motives, and can refer only to their publicly conveyed images and goal-settings.

On that level, however, we have clear and undisputable differences between the groups. The public image of the NGO leaders and the popes, i.e., their reputation in the public eye, is obviously more prosocial than (and as such even stands in opposition to) that of the DAX and DOW Jones CEOs, and so will be the success parameters according to which followers and members of the public judge them. Naturally, these factors matter for their selection, their performance, and their personal future. Consequentially, they will need to employ different problem-solving strategies and follow different behavioral conduct compared to leaders who openly pursue financial profits. Therefore, and in light of the fWHR literature, we find it intriguing that their faces, too, are wider than normal, with a deviation that is comparable in size to that of the investigated company’s CEOs. The result suggests that fWHR is generally higher in leaders across different (displayed) social value systems, from economically profit-seeking to (at least allegedly) altruistic and charitable. Note that by our reserved phrasing, we do not mean to cast doubt on the motivational integrity of any of the leaders of the non-profit seeking organizations. In fact, we hold the highest respect for some of these personalities and their work. However, as scientists we need to acknowledge the difference between evidence and presumption, and in this study, we have researched public portrayals, not inner motives.

At the same time, we note one other result showing that variables which we had originally interpreted in prosocial terms are actually not independent of financial success factors. As we analyzed the likability ratings of the DOW Jones CEOs more closely, we found that the associations between CEO’s fWHR and how much their companies and they themselves were liked by their employees were diminished when we controlled for company revenues. This again suggests intricate dependencies between business performance and social impact. In essence, a conglomerate of competitive motivation, strive for dominance, and social engagement appears to be associated with high fWHR, although our current design and sample size do not allow us to disentangle the relative contributions of these factors, or their interrelatedness. Larger samples enabling mediation analysis and experimental designs allowing for causal interpretation ought to shed light on this issue.

In our view, the remaining open questions do not lessen our demonstration of an empirical link between fWHR and social rank in public human life across nations, organizations, and value-systems. Overall, we have shown wider-than-normal faces in portraits of the world’s top leaders from different countries including Germany and the US, some operating in profit-seeking and competitively oriented contexts, others portraying themselves and their organizations as prosocial and charitable. We find this link between facial morphology and social image at the macro-level impressively illustrative of the ecological relevance of human fWHR. We stress that fWHR may not be the only facial feature that matters for selection of leaders [28], and welcome future attempts to disentangle the question of causality and the underlying mechanisms.

## Supporting information

S1 File Source data for all analyses. (XLSX)
